# *Cryptococcus deuterogattii* VGIIa Infection Associated with Travel to the Pacific Northwest Outbreak Region in an Anti-Granulocyte-Macrophage Colony-Stimulating Factor Autoantibody-Positive Patient in the United States

**DOI:** 10.1128/mBio.02733-18

**Published:** 2019-02-12

**Authors:** Shelly Applen Clancey, Emily J. Ciccone, Marco A. Coelho, Joie Davis, Li Ding, Renee Betancourt, Samuel Glaubiger, Yueh Lee, Steven M. Holland, Peter Gilligan, Julia Sung, Joseph Heitman

**Affiliations:** a Department of Molecular Genetics and Microbiology, Duke University Medical Center, Durham, North Carolina, USA; b Department of Medicine, University of North Carolina School of Medicine, Chapel Hill, North Carolina, USA; c Laboratory of Clinical Immunology and Microbiology, National Institute of Allergy and Infectious Diseases, National Institutes of Health, Bethesda, Maryland, USA; d Department of Pathology and Laboratory Medicine, University of North Carolina School of Medicine, Chapel Hill, North Carolina, USA; e Department of Radiology, University of North Carolina School of Medicine, Chapel Hill, North Carolina, USA; Albert Einstein College of Medicine; University of London; University of Aberdeen

**Keywords:** *Cryptococcus deuterogattii*, travel-acquired cryptococcal meningitis, anti-GM-CSF autoantibodies

## Abstract

The region encompassing the Pacific Northwest (PNW), Vancouver Island, Oregon, and Washington has been the location of an ongoing Cryptococcus gattii outbreak since the 1990s, and there is evidence that the outbreak is expanding along the West Coast into California. Here we report a clinical case of a 69-year-old, HIV-negative man from North Carolina who was diagnosed with a fungal brain mass by magnetic resonance imaging (MRI) and pathology. He had traveled to Seattle and Vancouver 3 years earlier and to Costa Rica 4 months prior to presentation. Phenotypic evidence showed that the fungal mass isolated from the patient’s brain represented C. gattii. In agreement with the phenotypic results, multilocus sequence typing (MLST) provided genotypic evidence that assigned the infecting organism within the C. gattii species complex and to the C. deuterogattii VGIIa clade. Whole-genome sequencing revealed >99.99% identity with the C. deuterogattii reference strain R265, indicating that the infecting strain is derived from the highly clonal outbreak strains in the PNW. We conclude that the patient acquired the C. gattii infection during his travel to the region 3 years prior and that the infection was dormant for an extended period of time before causing disease. The patient tested positive for anti-granulocyte-macrophage colony-stimulating factor (GM-CSF) autoantibodies, supporting earlier reports that implicate these autoantibodies as a risk factor associated with C. gattii infection.

## Abstract (precis)

Mortality rates associated with C. gattii infections are estimated to be between 13% and 33%, depending on an individual’s predisposition, and C. gattii has caused at least 39 deaths in the PNW region. There have been four other international travel cases reported in patients from Europe and Asia with travel history to the PNW, but this report describes the first North American traveler who acquired C. deuterogattii infection presenting within the United States and the first case of a C. deuterogattii outbreak infection associated with anti-GM-CSF autoantibodies. Early and accurate diagnoses are important for disease prevention and treatment and for control of infectious diseases. Continual reporting of C. deuterogattii infections is necessary to raise awareness of the ongoing outbreak in the PNW and to alert travelers and physicians to the areas of endemicity with potential risks.

## INTRODUCTION

Infectious diseases (e.g., tuberculosis and pneumonia) were the leading causes of death 150 years ago, but at the turn of the 21st century, the main cause of death changed from infectious diseases to physiopathological illnesses (e.g., heart disease, stroke, cancer) (1). Despite modern medical advances and the success of efforts to reduce the spread of infectious agents, infectious diseases still account for an estimated 13 million deaths per year compared to the approximately 40 million noninfectious deaths, the majority of which occur in developing countries (2, 3). Globalization and population expansion have increased the spread of infectious diseases despite strong efforts to counter these threats to public health. Additionally, international travel exposes tourists to a variety of diseases and infectious agents that they might not otherwise encounter, and with antibiotic and antifungal resistance in human pathogens on the rise, the ability to combat infectious diseases has become compromised (4, 5). With the increased mobilization of people, animals, and goods, early recognition among health care providers and the public is an essential component of preventing additional infectious disease cases.

*Cryptococcus* is a fungal pathogen responsible for nearly 220,000 human infections per year (6–8). Among the more than 30 *Cryptococcus* species, C. neoformans and C. gattii are responsible for the vast majority of human and animal infections. Unlike its sister species, C. neoformans, which is dispersed worldwide and primarily infects immunocompromised individuals, certain C. gattii subtypes often infect otherwise healthy subjects and are thought to have more restricted geographical niches (9). A 2014 study found that the presence of anti-granulocyte-macrophage colony-stimulating factor (GM-CSF) autoantibodies represented a risk factor for central nervous system infections of C. gattii but not C. neoformans, suggesting that previously unrecognized immune system defects may contribute to disease susceptibility (10). Both C. neoformans and C. gattii establish infection when desiccated yeast cells or spores are inhaled from the environment into the lungs, in some cases causing pneumonia, and then disseminate to the central nervous system to cause meningoencephalitis. The time from exposure of C. gattii to the onset of disease is thought to range from 2 to 12 months based on the reported cases of exposure but may be longer (11).

Historically, all C. gattii isolates have been classified as the same species but were separated into the following five different VG/AFLP molecular types: VGI, VGII, VGIII, VGIV, and AFLP10. New phylogenetic analyses and other data support the concept that all of the VG types actually represent different species: C. gattii (VGI), C. deuterogattii (VGII), C. bacillisporus (VGIII), C. tetragattii (VGIV), and C. decagattii (AFLP10) (12, 13). Using multilocus sequence typing (MLST), each lineage can be further divided into additional molecular subtypes. For example, the following multiple molecular subtypes exist in C. deuterogattii: VGIIa, VGIIb, and VGIIc. Notably, the *MAT***a** allele is underrepresented in all molecular types of the C. gattii species complex; the mating type locus (*MAT*) is a gene cluster containing alleles of one of two idiomorphs, *MAT***a** or *MAT*α, and determines mating compatibility (14). C. gattii and C. deuterogattii apparently have unequal numbers of *MAT***a** and *MAT*α isolates, and very few *MAT***a** strains of these species have been isolated from nature or from patients (15). In contrast, the *MAT***a** allele is more highly represented in the VGIIIc subtype of C. bacillisporus in that 39% of characterized strains are of the *MAT***a** mating type (16–18). It is important to characterize each lineage as its own species, as it has been shown that there is variability in ecological and host niches, virulence, and antifungal susceptibility, resulting in variable success in clinical treatment outcomes between the lineages (12).

C. gattii has long been a recognized pathogen in South America and Australia, and, more recently, an outbreak has been recognized on Vancouver Island, Canada, and in the United States Pacific Northwest (PNW), causing more than 300 reported human cases and at least 39 deaths, according to the Centers for Disease Control and Prevention (19). Within the last decade, numerous publications have reported genomic and phylogenetic analyses of the outbreak strains, advancing our understanding of the epidemiology and clinical associations of C. gattii (20, 21). C. deuterogattii has been identified as the causative agent for the ongoing epidemic in the PNW, and continuing human and veterinary cases confirm that the outbreak is spreading geographically down the West Coast (22, 23). C. deuterogattii is a threat not only to native inhabitants in the PNW and the surrounding areas (e.g., Washington and Oregon) but also to those who travel to the area. In fact, travel-related cases have been reported since the outbreak began (24–28), but the cases appear to represent international tourists from Europe and Asia visiting the PNW.

Here we present a case of travel-related cryptococcal central nervous system (CNS) disease in which an individual residing in North Carolina acquired a C. deuterogattii infection during travel to the PNW but did not begin to show symptoms until several years after returning home. Unique to this case is the extended incubation period of C. deuterogattii, which was three times longer than previously predicted latency periods, before causing disease. Additionally, the patient tested positive for anti-GM-CSF autoantibodies, a suggested risk factor for C. gattii infections (10, 29). Although there have been reports of international travel to the PNW resulting in C. deuterogattii infections (25, 26, 28), to our knowledge this is the first reported case of a travel-associated cryptococcal CNS disease caused by C. deuterogattii from the Pacific Northwest presenting in the United States. Globalization continues to influence the emergence and resurgence of infectious diseases, and continual reporting of travel-related cases of C. deuterogattii is crucial to raise awareness of the ongoing fungal outbreak in the PNW and potential risks to travelers. Additionally, health providers treating domestic patients should be aware of their travel history, even in cases of remote travel years prior to presentation.

## RESULTS

### Clinical presentation and treatment.

**FIG 1:**
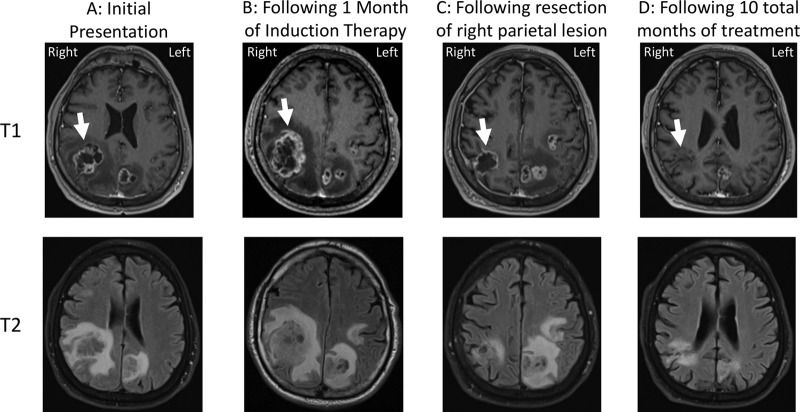
Changes in parietal lobe lesions on brain MRI over the course of treatment. The top panels represent post-gadolinium T1-weighted images, and the bottom panels represent post-gadolinium T2-weighted images. (A) MRI at initial presentation. Circumferential ring enhancement on the post-gadolinium T1-weighted axial image and increased signal surrounding the lesion on the axial T2-fluid attenuation inversion recovery (FLAIR) image represent marked vasogenic edema (arrow). The heterogeneously increased signal within the cavity of the lesions represents necrosis. (B) MRI following 1 month of induction therapy. Increased T2 signal within the lesion cavity on the FLAIR sequence and increased surrounding edema and the overall size of the right parietal lesion indicate an interval of worsening with likely progressive necrosis of brain tissue (arrow). A leftward midline shift was noted. (C) MRI following resection of the right parietal lesion. Note improvement in the edema surrounding the lesion on the FLAIR sequence and an interval of decrease in the size of the right parietal lesion (arrow). (D) Subsequent MRI imaging after a total of 10 months of treatment. Improved T2/FLAIR signal abnormality with a decrease in the size of the resection cavity (arrow) as well as decrease in the size of the other visible lesions was observed.

The patient was a 69-year-old man who presented with 3 weeks of clumsiness, dizziness, shuffling gait, memory trouble, headache, and worsening fine motor skills. He reported a cough and shortness of breath for 1 to 2 months that had resolved 3 months prior to presentation and had just returned from an intense bicycling trip that had lasted several days. Because of the new and progressive neurological symptoms, magnetic resonance imaging (MRI) of the brain with contrast was performed and showed multiple ring-enhancing lesions with associated edema and central necrosis (Fig. 1A). The largest lesion (3.7 by 3.1 cm) was in the right parietal lobe (Fig. 1A, white arrow), with a 3-mm leftward midline shift. Other abnormalities were present in the left inferior temporal lobe (2.2 by 1.8 cm), frontoparietal lobe (1.7 by 1.7 cm), and parietal lobe (2.3 by 2.0 cm), as well as along the midline dura (2.4 by 1.0 cm).

**FIG 2:**
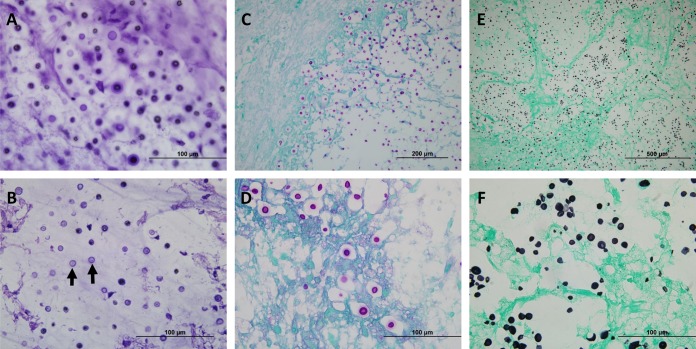
Cryptococcal organisms can be visualized within the brain parenchyma of both intraoperative and fixed tissues. (A) Intraoperative touch preparation/smear depicting classic “soap bubble” lesions comprised of yeast cells within clear gelatinous capsules infiltrating the brain parenchyma. An absence of inflammatory cells was noted; the lack of an appreciable inflammatory response is characteristic of many cryptococcal infections. The scale bar represents 100 μm. (B) An intraoperative frozen section demonstrated spherical-to-oval, variably sized, encapsulated yeast cells. The organisms were refractile, and many had circumferential halos. The scale bar represents 100 μm. (C) Low-magnification image of periodic acid-Schiff (PAS) staining showing infiltration of brain parenchyma by positively staining yeast cells, morphologically consistent with *Cryptococcus*. The scale bar represents 200 μm. (D) Higher magnification of the image in panel C highlighting the refractile appearance and the intensely stained mucopolysaccharide capsule of the yeast forms in tissue sections. Of note, mucicarmine and alcian blue also showed positive staining in the capsule of cryptococcal organisms (not shown). The scale bar represents 100 μm. (E) Low-magnification image of Grocott-Gomori’s methenamine silver (GMS) staining showing a representative field with innumerable yeast cells, some with narrow-based budding. The scale bar represents 500 μm. (F) Higher magnification of the image in panel E illustrating the intense staining of organisms within the brain parenchyma (stained light green by the counterstain). The scale bar represents 100 μm.

Given concern that the lesions represented metastases, computed tomography of the chest, abdomen, and pelvis was performed, revealing a 2-cm, spiculated right lower lobe nodule consistent in appearance with small cell lung cancer. The lung lesion was not amenable to sampling, so the left temporal lobe mass from the brain was partially resected for definitive diagnosis. Intraoperatively, a frozen section of the firm, gray-colored mass revealed fungal organisms (Fig. 2B). Following a review of the patient’s travel history that revealed a trip to Costa Rica 4 months prior to symptom onset where he visited a bird sanctuary, as well as a trip to Vancouver and Seattle 3 years prior lasting several weeks, patient serum was sent for cryptococcal antigen testing and returned positive results, with a titer of 1:1,280 (Table 1). The patient was started on liposomal amphotericin B (AmB) at 4 mg/kg of body weight daily combined with 2,000 mg of 5-flucytosine (5-FC) four times daily as induction therapy for a presumed cryptococcoma. Additionally, an extended dexamethasone taper was prescribed for cerebral edema (4 mg twice daily for 6 days; the dose was then halved every 3 days for a total of an 18-day course).

**TABLE 1:** Patient laboratory results^*a*^

Parameter	Value(s)			
	Normal range	At time of diagnosis	After 4 wks of induction therapy	After 8 wks of induction therapy
Serum				
Cryptococcal antigen titer	Negative	1:1,280	1:320	1:2,560
Creatinine	0.7–1.30 mg/dl	0.72	1.73	1.59
AST	19–55 U/liter	33	59	27
ALT	19–72 U/liter	31	123	<8
WBC	4.5 × 10^9^–11 × 10^9^ cells/liter	13.3	3.9	4.5
Platelets	150 × 10^9^–440 × 10^9^ cells/liter	254	140	174
ANC	2.0 × 10^9^–7.5 × 10^9^ cells/liter	12.2	1.9	2.2
ALC	1.5 × 10^9^–5.0 × 10^9^ cells/liter	0.6	1.3	1.7
Absolute CD4 count (%)	510–2,230 cells/µl (34%–58%)	228^*b*^ (38%)	871 (56%)	
Absolute CD8 count (%)	180–1,520 cells/µl (18%–38%)	132^*b*^ (22%)	358 (23%)	

CSF				
Opening pressure		27 mm Hg	ND	ND
Cryptococcal antigen	Negative	1:40	ND	ND
Culture	Negative	Negative	ND	ND
Nucleated cells	0–5/μl	5	ND	ND
Protein	15–45 mg/dl	48 mg/dl	ND	ND
Glucose	50–75 mg/dl	75 mg/dl	ND	ND

a ND, not determined. Data represent patient serum and cerebrospinal fluid (CSF) laboratory results at the time of diagnosis and 4 and 8 weeks after induction therapy. AST, aspartate aminotransferase; ALT, alanine transaminase; WBC, white blood cell count; ANC, absolute neutrophil count; ALC, absolute lymphocyte count.

b Testing was done three days after initiation of dexamethasone treatment.

A culture of the brain mass turned positive on day 3 of incubation. Matrix-assisted laser desorption–ionization time of flight (MALDI-TOF) analysis of the presumed fungal organism revealed a spectrum consistent with C. gattii. A lumbar puncture was performed immediately, and cerebral spinal fluid (CSF) was sent for cryptococcal antigen testing. This returned a positive (1:40) result, although culture of the CSF was negative (Table 1). Fourth-generation testing for HIV was negative, and the CD4^+^ T cell count obtained once the patient had completed the dexamethasone taper was normal at 871. Hemoglobin A1c was also within the normal range. Anti-GM-CSF testing was performed on the patient’s plasma and detected high titers of these autoantibodies; in addition, elevated levels of the immune system components interferon gamma (IFN-γ) and interleukin-17A (IL-17A) were noted (Table 2).

**TABLE 2:** Anti-GM-CSF autoantibody screening^*a*^

Sample	RLU value(s)		
	IFN-γ	IL-17A	Anti-GM-CSF autoantibodies
Healthy control (*n* = 4)	217 ± 263	367 ± 461	59 ± 46
Positive control	18,085	12,850.5	5,397.5
Patient	733.8	731.3	4,711.3

a Screening of patient plasma for interferon gamma (INF-γ), interleukin-17A (IL-17A), and anti-granulocyte-macrophage colony-stimulating factor (GM-CSF) autoantibodies. The positive-control data represent other patients with demonstrated titers and disease, while negative-control data represent healthy controls from a blood blank. The reported values represent measures of fluorescence intensity (relative light units [RLU]).

The patient improved clinically over the next 4 weeks of induction therapy but then developed mild acute kidney injury (AKI) and cytopenia, prompting discontinuation of AmB and 5-FC treatment and transition to the renally dosed equivalent of 800 mg fluconazole daily. Two days later, a repeat MRI of the brain was done to assess response to induction treatment and revealed an increase in the size of the right parietal lesion and significant vasogenic edema (Fig. 1B). AmB treatment was then restarted and administered in addition to the fluconazole treatment, and a resection of the enlarging parietal lesion was performed. Pathology demonstrated a cryptococcoma with Grocott-Gomori’s methenamine silver (GMS) stain positive for fungal elements morphologically consistent with *Cryptococcus*. No culture was performed at the time of the second resection. The patient received an additional 48 h of dexamethasone postoperatively, but as he was otherwise clinically stable, no further steroid treatment was prescribed.

At 2 weeks later, after improvement in the cytopenia results, fluconazole treatment was stopped and the patient was restarted on 5-FC with continuation of AmB. Over the following 2 weeks, the patient demonstrated continued slow improvement in symptoms, and brain MRI imaging showed stability of lesions (Fig. 1C). Therefore, he was again transitioned to consolidation therapy with a renally equivalent dose of 800 mg fluconazole daily. After 12 additional weeks of high-dose fluconazole, maintenance therapy was started with fluconazole 400 mg daily. His neurological symptoms gradually improved, with resolution of gait abnormalities and continued improvement in strength and coordination. As expected, the AKI and cytopenias resolved with cessation of the AmB and 5-FC. A follow-up brain MRI completed after approximately 10 months of treatment revealed significant improvement (Fig. 1D). Prolonged therapy with fluconazole was anticipated until achievement of complete resolution of the remaining intracranial lesions. A detailed time line of the patient presentation, resections, and antifungal treatment is shown in Fig. S1 in the supplemental material.

10.1128/mBio.02733-18.1 FIG S1 Detailed time line of the patient’s clinical course. Download FIG S1, TIF file, 0.01 MB. Copyright © 2019 Clancey et al. 2019 Clancey et al. This content is distributed under the terms of the Creative Commons Attribution 4.0 International license.

### Phenotypic analysis placed the clinical isolate in the C. gattii species complex.

**FIG 3:**
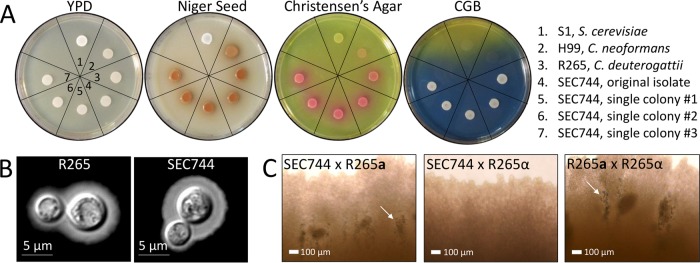
The clinical isolate displays phenotypes consistent with a C. gattii *MAT*α isolate. (A) Phenotypic testing of the isolate on YPD; niger seed; Christensen’s agar; and canavanine, glycine, and bromothymol blue (CGB) media. Section 1, S1 (Saccharomyces cerevisiae); section 2, H99 (C. neoformans); section 3, R265 (C. deuterogattii); section 4, clinical isolate thick streak (SEC744); section 5, clinical isolate single colony 1; section 6, clinical isolate single colony 2; section 7, clinical isolate single colony 3. (B) Capsule staining with India ink. Scale bars represent 5 μm. (C) Imaging of mating patches of the clinical isolate (SEC744) tested against R265 *MAT***a** and R265 *MAT*α. R265 *MAT***a** × *MAT*α served as a positive control. Arrows indicate mating structures visible by light microscopy at ×20 magnification. Scale bars represent 100 μm.

To confirm the MALDI-TOF results that characterized the clinical isolate as C. gattii, the isolate was subjected to a number of phenotypic tests. The isolate was grown on media to test for growth (YPD); melanin production (niger seed); urease production (Christensen’s agar); and growth and color development on medium containing canavanine, glycine, and bromothymol blue (CGB). The isolate produced dark pigment on niger seed (melanin) and pink coloration on Christensen’s agar (urease); both results are consistent with all pathogenic *Cryptococcus* species (Fig. 3A). The isolate grew and produced blue pigment on CGB agar, which is unique to C. gattii species (Fig. 3A) (30). *Cryptococcus* produces a hydrophilic extracellular capsule as a means of evading the host immune system (31). To analyze capsule production, India ink staining and microscopic evaluation were performed after growth in CO_2_-independent medium for 72 h at 37°C. Using the C. deuterogattii R265 reference strain for comparison, the patient isolate produced similar capsules (Fig. 3B).

As previously mentioned, the majority of isolates characterized in C. gattii are of the *MAT*α mating type. To determine the mating type of the clinical isolate, it was cocultured with known C. deuterogattii VGIIa R265 *MAT***a** and *MAT*α isolates. The clinical isolate produced mating structures (hyphae, basidia, and spores) only when paired with a *MAT***a** partner, indicating that the isolate was of the α mating type (Fig. 3C). No self-sporulation was observed when the patient isolate was incubated alone on mating media, providing evidence that the isolate was not an **a-**α diploid or self-fertile (Fig. S2).

10.1128/mBio.02733-18.2 FIG S2 The clinical isolate did not sporulate in solo culture. Clinical isolate SEC744, R265 *MAT*α, and R265 *MAT***a** were patched individually onto mating media (V8; pH = 5). No hyphae or spore formation were observed. Download FIG S2, TIF file, 0.4 MB. Copyright © 2019 Clancey et al. 2019 Clancey et al. This content is distributed under the terms of the Creative Commons Attribution 4.0 International license.

### Molecular analysis placed the clinical isolate in the VGIIa major clade.

**FIG 4:**
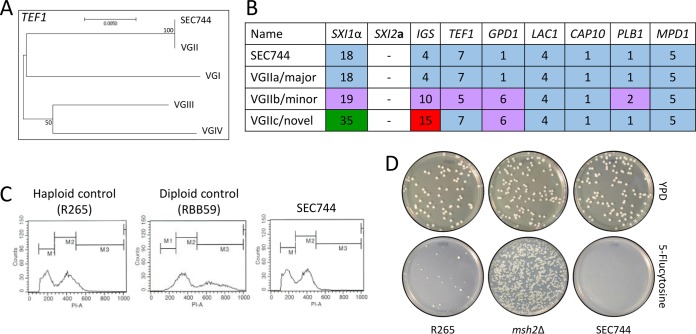
Genotyping places the clinical isolate in the C. deuterogattii VGIIa major lineage. (A) Representative MLST tree of *TEF1*. The scale bar represents the number of substitutions per site. (B) Allele designations for the SEC744 clinical isolate and the C. deuterogattii VGIIa, VGIIb, and VGIIc lineages. (C) FACS analysis of C. deuterogattii R265 (haploid control), RBB59 (C. gattii diploid), and the SEC744 clinical isolate. PI-A, propidium iodide area. (D) Testing for hypermutator phenotype in R265, the *msh2*Δ mutant, and the SEC744 clinical isolate.

MLST analysis was performed to assign the clinical isolate to a species within the C. gattii species complex. MLST loci were PCR amplified with primers designed to amplify each sequence, the PCR products were purified and analyzed by Sanger sequencing, and the sequences were assembled in Sequencher using forward and reverse strands to ensure complete double-stranded coverage. Phylogenetic analysis was performed using representative sequences from each of the C. gattii molecular types: C. gattii (VGI), C. deuterogattii (VGII), C. bacillisporus (VGIII), and C. tetragattii (VGIV). The gene sequences of the clinical isolate clustered closely with the C. deuterogattii representative sequence, supported with high bootstrap values (Fig. 4A; see also Fig. S3). BLAST analysis of the contigs from each locus showed 100% sequence identity to the same loci in VGIIa sequences already deposited in NCBI. Allele designations were assigned for each of the loci, resulting in a clear pattern of the VGIIa genotype (Fig. 4B). Taken together, the genotyping data assign this clinical isolate to the VGIIa major lineage of C. deuterogattii. Fluorescence-activated cell sorter (FACS) analysis was employed to determine that the isolate is haploid, containing a single copy of each chromosome (Fig. 4C).

10.1128/mBio.02733-18.3 FIG S3 Phylogenetic analysis of six loci in the clinical isolate cluster with C. deuterogattii. MLST trees corresponding to *IGS*, *LAC1*, *GPD1*, *CAP10*, *PLB1*, and *MPD1* show that these gene sequences are all more similar to the same loci in C. deuterogattii than to those in C. gattii (VGI), C. deuterogattii (VGII), C. bacillisporus (VGIII), and C. tetragattii (VGIV). All results are supported by strong bootstrap values. The scale bar represents the number of substitutions per site. Download FIG S3, TIF file, 0.1 MB. Copyright © 2019 Clancey et al. 2019 Clancey et al. This content is distributed under the terms of the Creative Commons Attribution 4.0 International license.

A lineage of C. deuterogattii VGIIa-like isolates was previously described that displayed a hypermutator phenotype resulting from a loss-of-function mutation in the *MSH2* gene, encoding the MutS homolog required for mismatch DNA repair (32). Hypermutator strains display increased resistance to the fluorinated orotic acid derivative 5-fluoroorotic acid (5-FOA), the immunosuppressant FK506, and the clinically relevant antifungal drug 5-FC, suggesting a mode of resistance to antifungal therapy (32). To determine whether the clinical isolate described here would exhibit the same phenotype, the isolate was exposed to 5-FC and the ability of the strain to produce 5-FC-resistant colonies was compared to the activity seen with the wild-type and hypermutator control strains. Unlike the *de novo msh2*Δ mutant, which produced a large number of 5-FC-resistant colonies, the clinical isolate did not produce any colonies on medium supplemented with 5-FC (Fig. 4D). This observation shows that the isolate displayed a largely wild-type level of intrinsic mutation and is not a member of the hypermutator lineage.

### The clinical isolate was shown to share its origin with C. deuterogattii outbreak strains.

**FIG 5:**
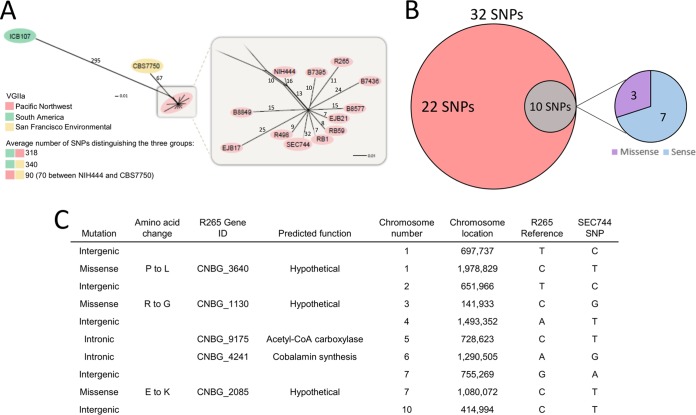
Phylogenetic analysis suggests that the clinical isolate originated in the PNW. (A) Phylogenetic tree depicting association of the SEC744 clinical isolate with other C. deuterogattii VGIIa outbreak strains from the PNW. Numbers on branches represent the number of SNPs distinguishing the groups. (B) Venn diagram illustrating the SNPs found in the SEC744 clinical isolate compared to the outbreak VGIIa R265 reference strain. (C) Table classifying the location and nature of the 10 private SNPs in the SEC744 clinical isolate. CoA, coenzyme A; ID, identifier.

The isolate was subjected to whole-genome sequencing, and the resulting sequence was used in phylogenetic analysis performed with other previously described C. deuterogattii outbreak strains from the PNW (15). The clinical isolate clustered with 10 highly clonal outbreak strains, including the VGIIa major reference strain R265 (Fig. 5A). In accord with the phenotypic data, it did not fall within the hypermutator lineage that includes NIH444, CBS7750, and ICB107 (32). Alignment of the clinical isolate to the R265 reference strain revealed a total of 32 single-nucleotide polymorphisms (SNPs) that differed between the two strains. Among these SNPs, 22 were shared with at least one other strain in the outbreak group whereas 10 SNPs were unique to the clinical isolate (Fig. 5B). Of the 10 private SNPs, 7 were intergenic or intronic mutations and 3 were missense mutations that resulted in an amino acid change in the protein coding sequence. These nonsynonymous amino acid substitutions were all in uncharacterized genes and likely had no impact on the patient’s response to clinical treatment (Fig. 5C).

## DISCUSSION

In this report, we document the first case of a previously healthy individual becoming infected with C. deuterogattii after travel from the United States to the PNW outbreak region. Three largely clonal groups of isolates of C. deuterogattii have been identified circulating within the PNW: VGIIa/major, which causes the majority of human and animal infections; VGIIb/minor, which causes fewer infections; and VGIIc/novel, which thus far has been reported only from Oregon and not from Seattle or Canada (21). More recent studies have suggested that C. gattii originated in South America, where it is endemic, which makes the outbreak in the PNW exceptional, given the temperate climate (33, 34). A 2018 study by Roe and colleagues estimated C. gattii was introduced to the PNW between 60 and 100 years ago, coinciding with the completion of the Panama Canal and the expansion of international shipping from South America (35). However, C. deuterogattii VGIIb more closely aligns with isolates from Australia, suggesting multiple possible routes, sources, and introductions. A report from 2009 by Byrnes and colleagues documented a case of a travel-acquired C. gattii (VGI) infection (36); since then, four international travel-related C. deuterogattii (VGII) cases in individuals from Denmark, The Netherlands, Germany, and Japan have been reported (25–28). Overall, there has been a noticeable increase in the number of travel-related cases being reported. Combined with the C. deuterogattii infection described here, this continues to elevate the level of attention to cryptococcal infections.

Following clinical presentation, a fungal mass was removed from the brain of the patient and subjected to phenotypic and genotypic analyses. The patient fungal isolate was identified as C. deuterogattii by the use of microbiological techniques, and detailed MLST and whole-genome sequencing confirmed this finding. Further phylogenetic analysis showed that the patient isolate clusters closely with previously described PNW outbreak strains (15). Among the strains identified as part of the PNW outbreak, three were characterized as being hypermutators due to their ability to produce drug-resistant colonies at a high frequency. Following exposure to the clinically relevant compound 5-FC, the patient isolate did not produce a large number of drug-resistant colonies, suggesting that it is not a member of this hypermutator lineage (32). Whole-genome sequencing revealed 32 SNPs differentiating the patient isolate from the R265 reference strain. Among those SNPs, 22 were also found in at least one outbreak strain, but the clinical isolate contained 10 unique SNPs. The private SNPs do not appear to have influenced the pathogenicity of the infecting strain or treatment outcome, as most are intergenic or resulted in amino acid changes in uncharacterized hypothetical proteins unlikely to play a significant role in disease (Fig. 5).

Taking the data together, the clustering of the patient sample with the 10 other highly clonal outbreak strains and the absence of a hypermutator phenotype provide strong evidence that the patient isolate originated within the PNW and that the patient had acquired the infection during his visit to the area 3 years prior, which laid dormant until symptoms began following the return of the patient to North Carolina. To our knowledge, this represents the longest incubation period for a human C. gattii infection to have been reported in the literature. However, there is currently limited evidence available to support an accurate estimate. Review of cases from the outbreak in the PNW suggests an incubation period of 2 to 11 months, with a median of 6 to 7 months (11). Although the patient also had a history of travel to a bird sanctuary in Costa Rica 4 months prior to the onset of symptoms, this seems less likely to have been the source of infection, as C. deuterogattii VGIIa infection is not known to be endemic in that area.

Cryptococcal meningitis in healthy individuals is somewhat rare (1 in 100,000), but the presentation of such cases suggests that normal hosts could harbor rare primary immunodeficiencies (37). Cryptococcal meningitis has been associated with a number of iatrogenic and inherited immunosuppressive conditions, including organ transplantation, hematopoietic malignancies, and steroid use. Furthermore, a number of defects in cell-mediated immunity have been identified as predisposing factors for cryptococcal meningitis. The most common immunodeficiency in non-HIV cryptococcal meningitis is idiopathic CD4^+^ lymphopenia, a disease caused by various alleles, which is seen in over 25% of cases (37). Additionally, the presence of anti-GM-CSF and IFN-γ autoantibodies has also been associated with cryptococcal disease (10, 38). Monogenic mutations, such as those in the zinc transcription factor *GATA2* gene, the transcriptional activator *STAT3* gene, and the CD40 ligand *CD40LG* gene, are also associated with cryptococcosis in seronegative HIV patients (39–41). The patient described in this study did not have any documented iatrogenic causes of immunosuppression or history of immunodeficiency. Hyper-IgM syndromes often present earlier in life, i.e., in infancy or childhood, manifesting as increased susceptibility to sinopulmonary and opportunistic infections; this patient did not have such a history. Whole-genome sequencing or exome sequencing of the patient could have determined if any of these underlying non-HIV-related predisposing factors played a role in the patient’s acquisition of disease, but such analyses were not performed in this study. Additionally, there are data to suggest that total and memory B-cell levels are lower in HIV-uninfected patients with C. neoformans infections (42). However, only limited flow cytometry measuring T-cell but not B-cell populations was performed in this case.

Interestingly, analysis of the patient’s blood did show high titers of anti-GM-CSF autoantibodies, which are known to cause acquired pulmonary alveolar proteinosis (PAP) through dysregulation of the function of macrophages, a cell type important for the control of cryptococcal infection. Specifically, these antibodies block phosphorylation of STAT5, which inhibits macrophage signaling (10, 29). Biologically inhibitory anti-GM-CSF autoantibodies have been associated with cryptococcal meningitis in some otherwise immunocompetent patients and in those with idiopathic CD4^+^ lymphopenia. However, it has yet to be confirmed whether they are induced by an unknown trigger, preceding and therefore increasing susceptibility to infection, or whether they are produced in response to infection. A notable absence of high-titer anti-GM-CSF autoantibodies has been found in the vast majority of healthy and immunodeficient control patients without cryptococcal disease reported in the literature thus far (29). In contrast, a recent study did find one healthy control who had anti-GM-CSF antibodies. His plasma inhibited STAT5 production but to a much lesser degree than is seen with those with cryptococcal infection and autoantibodies (10). Because screening for anti-GM-CSF autoantibodies is not routinely performed in healthy individuals, it is not possible to know whether the autoantibodies predated or followed the acquisition of C. deuterogattii by the patient described in this case. The patient was screened for PAP in the clinical evaluation, but a bronchoscopy was not performed as his symptoms did not warrant it (i.e., no progressive dyspenea on exertion, chronic cough, weight loss, or fatigue). As of 15 months after the initial diagnosis, he had not had any symptoms or computed tomography (CT) chest imaging results consistent with PAP.

Among HIV-uninfected patients, intracranial infection caused by C. gattii is associated with a delayed response to therapy and with a higher incidence of neurosurgical intervention than is associated with C. neoformans infection (43). In all patients, C. gattii infection is more likely to cause intracranial cryptococcomas, which often respond poorly to antifungal treatment. Guidelines recommend the same forms of induction, consolidation, and suppressive treatment for infections due to both cryptococcal species, with extension of induction therapy to 6 weeks in those with cryptococcomas. In addition, surgery should be considered for lesions ≥3 cm in diameter with mass effect or for those that fail to reduce in size after 4 weeks of therapy or if there is compression of vital structures (2010 Infectious Diseases Society of America [IDSA] guidelines). Use of adjunctive corticosteroids in the setting of mass effect or vasogenic edema is supported by moderate evidence, but it is of limited (level III) quality and is given weak recommendations. The patient described here showed initial clinical improvement in response to partial resection, steroids, and 4 weeks of induction therapy but demonstrated radiographic deterioration with enlargement of the lesions and surrounding edema. This could have been due to completion of the steroid taper (the last dose was administered 9 days prior to MRI results demonstrating increased sizes of lesions), incomplete response to induction therapy (especially in the setting of potential immunodeficiency), or an immune reconstitution inflammatory response-like syndrome such as has been reported during treatment of C. gattii infection (44). While pathology analysis did reveal fungal elements morphologically consistent with *Cryptococcus*, the GMS stain does not differentiate between live and dead organisms. Unfortunately, at the time of repeat resection, no culture of the lesion was done to determine if radiographic worsening was due to sterile inflammation or to persistent infection. Since the second neurosurgical procedure, the patient has continued to recover clinically and has also demonstrated significant radiological improvement. While he did receive 2 days of steroids postoperatively, it is unlikely that this would have resulted in the gradual, sustained improvement that ultimately followed. The slow progress suggests that although the second debulking surgery likely played a key role in his management, prolonged antifungal therapy was crucial to his treatment.

Disease caused by C. gattii can differ from that of C. neoformans based on host risks and clinical presentation. In this clinical case, a previously immunocompetent male presented with brain lesions, tested positive for anti-GM-CSF antibodies, and responded relatively well to treatment. Due to the extended (∼3-year) dormancy stage of this infecting strain, detailed travel history was key in determining the cause and geographical source of the brain lesion. The need for detailed travel history with heightened clinical suspicion is critical, as the hosts in which C. gattii presents often differ from those in which C. neoformans presents. This case represents another example of the presence of anti-GM-CSF autoantibodies in a host concurrent with C. gattii infection, further strengthening the argument for anti-GM-CSF autoantibodies as a risk factor for C. gattii infection. Increased awareness of this factor will enhance the identification of diseases by treating physicians. In turn, this will lead to treatments that are more appropriate, precise, and timely and, ultimately, to reduced morbidity and mortality.

## MATERIALS AND METHODS

### Magnetic resonance imaging (MRI).

Imaging was performed using gadolinium contrast medium.

### Pathology.

Intraoperative smear and frozen sectioning was performed at ×60 magnification using hematoxylin and eosin. Periodic acid-Schiff (PAS) staining and Grocott-Gomori’s methenamine silver (GMS) staining were performed at ×20 and ×60 magnification using formalin-fixed, paraffin-embedded tissue.

### Phenotypic tests.

Strains were grown overnight in liquid YPD (1% yeast, 2% peptone, 2% dextrose), washed with sterile distilled water (dH_2_O), and spotted onto solid YPD agar (1% yeast, 2% peptone, 2% dextrose, 1.5% agar); niger seed agar (7% niger seed, 0.1% glucose, 2% agar); Christensen’s agar (0.1% gelatin peptone, 0.1% dextrose, 0.012 g phenol red, 0.2% KH_2_PO_4_, 1.2% agar); and canavanine, glycine, and bromothymol blue (CGB) agar (10% solution A [1% glycine, 0.1% KH_2_PO_4_, 0.1% MgSO_4_, 1 mg thiamine-HCl, 30 mg l-canavanine sulfate], 2% solution B [0.08% sodium bromothymol blue], 2% agar). Plates were incubated at 30°C for 2 to 3 days.

### Capsule staining.

The R265 control strain and the SEC744 clinical isolate were inoculated into liquid YPD (1% yeast, 2% peptone, 2% dextrose) and grown overnight at 30°C with rotation. Cultures were diluted 1:10 into 5 ml of CO_2_-independent medium and incubated for 3 days at 37°C with rotation. A 500-μl volume of cells was pelleted and resuspended in 50 μl in sterile water. Following resuspension, a 1.5-μl volume of cells was spotted onto a microscope slide along with 1 μl of India ink. Capsule staining was visualized using bright-field microscopy on a Zeiss Axioskop 2 Plus microscope.

### Mating assay.

Using sterile toothpicks, the SEC744 clinical isolate and R265 *MAT*α and R265 *MAT***a** were patched together onto V8 (pH 5; 5% V8 juice, 0.05% KH_2_PO_4_, 4% agar) as shown in Fig. 2B. Mating plates were incubated in the dark at room temperature for 1 month, and mating patches were observed under a Nikon Eclipse E400 microscope at ×40 magnification.

### Phylogenetic analysis.

Genes were amplified under typical PCR conditions using primers listed in Table S1 in the supplemental material. PCR products were purified and sent for Sanger sequencing (Genewiz, Research Triangle Park, NC). Consensus sequences were built using Sequencher with forward and reverse strands. Gene phylogenies were built using Mega7 software with bootstrap values of 100 and the maximum likelihood method based on the Tamura-Nei model (45). Representative consensus sequences for VGI, VGII, VGIII, and VGIV were acquired from GenBank entries.

10.1128/mBio.02733-18.4 TABLE S1 Primers used in this study. Download Table S1, DOCX file, 0.01 MB. Copyright © 2019 Clancey et al. 2019 Clancey et al. This content is distributed under the terms of the Creative Commons Attribution 4.0 International license.

### Multi-locus sequence typing (MLST).

To determine the molecular subtype of the SEC744 clinical isolate, we aligned the consensus sequence from the phylogenetic analysis (Fig. 4A; see also Fig. S3 in the supplemental material) to GenBank entries using Mega7 software. In cases in which a 100% homologous alignment was found, the allele was assigned with the already established allele designation numbers described by Fraser et al. (46).

### Fluorescence-activated cell sorter (FACS) analysis.

Actively growing cells were scraped from a YPD agar plate (1% yeast, 2% peptone, 2% dextrose, 1.5% agar), washed with 1 ml phosphate-buffered saline (PBS), and fixed overnight at 4°C in 1 ml 70% ethanol. Cells were then washed with 1 ml NS buffer (10 mM Tris-HCl [pH 7.6], 250 mM sucrose, 1 mM EDTA [pH 8.0], 1 mM MgCl_2_, 0.1 mM CaCl_2_, 0.1 mM ZnCl_2_, 0.4 mM phenylmethylsulfonyl fluoride, 7 mM β-mercaptoethanol) and then resuspended in 180 μl NS buffer with 5 μl propidium iodide (0.5 mg/ml) and 20 μl RNase (10 mg/ml). Cells were stained overnight at 4°C. Prior to analysis on a flow cytometer, 50 μl of stained cells was diluted into 2 ml of 50 mM Tris-HCl (pH 8.0) in a 5-ml Falcon tube. Flow cytometry was performed on 10,000 cells, and the results were analyzed on the FL1 channel on a Becton, Dickinson FACScan apparatus.

### Hypermutator assay.

Single colonies were inoculated into a 5-ml overnight liquid culture with YPD (1% yeast, 2% peptone, 2% dextrose). Cells were pelleted, washed with 5 ml dH_2_O, and resuspended in 5 ml dH_2_O. A 100-μl volume of a 10^−5^ dilution was plated on YPD agar (1% yeast, 2% peptone, 2% dextrose, 1.5% agar) to check cell viability, and 100 μl of undiluted culture was plated on YNB–5-FC (0.67% yeast nitrogen base, 2% glucose, 2% agar, 100 μg/ml 5-FC).

### DNA extraction and genome sequencing.

Genomic DNA was extracted using cetyltrimethylammonium bromide (CTAB) buffer. A 25-ml culture of the SEC744 isolate was pelleted and lyophilized before pulverization with glass beads was performed. The dried cell debris was resuspended in 10 ml CTAB buffer and heated at 65°C for 10 min. An equal volume of chloroform was added to the mixture, and the solution was gently mixed. Following centrifugation, the aqueous phase was transferred to a new tube and DNA was precipitated using isopropanol. The DNA was pelleted, resuspended in dH_2_O, and treated with 20 μg/ml of RNase A. Whole-genome sequencing was performed at the Duke University Sequencing and Genomic Technologies Shared Resource (Durham, North Carolina). Libraries were prepared using a Kapa Hyper Prep Kit, and the libraries were run on an Illumina Hi-Seq 4000 instrument to generate 150-bp paired-end reads.

### SNP calling and phylogenetic networks analysis.

The phylogenetic relationship of the SEC744 clinical isolate to other strains of the C. deuterogattii VGIIa subpopulation was inferred from a matrix containing the SNP calls for the selected set of samples. The genomic paired-end reads were mapped to the C. deuterogattii R265 reference genome (GenBank accession no. GCA_002954075.1) (47) using BWA-MEM short read aligner v.0.7.17-r1188 (48) with default settings. SNP discovery, variant evaluation, and further refinements were carried out with the Genome Analysis Toolkit (GATK) best practices pipeline (49) v.4.0.1.2, including the use of Picard tools to convert SAM to sorted BAM files, fix read groups (module: AddOrReplaceReadGroups; SORT_ORDER=coordinate), and mark duplicates. Variant sites were identified with the HaplotypeCaller from GATK using the haploid mode setting and were subsequently filtered using the VariantFiltration module with the following criteria: QD < 2.0 ǁ FS > 60.0 ǁ MQ < 40.0 ǁ MQRankSum < -12.5 ǁ ReadPosRankSum < -8.0 ǁ SOR > 4.0. Next, variants were parsed with VCFtools v0.1.16 (50) to include only high-quality SNPs, here defined as SNPs with a mean depth of >10 (–minDP 10) and a minimum quality of 30 (–minQ 30). The option –max-missing 1 was used to exclude sites with missing data in at least one sample. The resulting VCF files were parsed with a custom Perl script into a concatenated FASTA consisting of 526 sites and were then imported to SplitTree v.4.14.2 (51) to construct an unrooted split-network using the Neighbor-net method. The potential mutational effect of the 10 private variants found in the SEC744 clinical isolate was manually inspected by substituting the corresponding bases in the annotated genes of C. deuterogattii R265 with these variants.

### Anti-granulocyte-macrophage colony-stimulating factor (GM-CSF) autoantibody testing.

Anti-GM-CSF autoantibody testing was performed at the National Institute of Allergy and Infectious Diseases in Bethesda, MD. Briefly, the ability of the antibody to directly bind to interferon gamma (INF-γ), interleukin-17A (IL-17A), or anti-GM-CSF autoantibody was assessed and measured as a function of fluorescence intensity (52).

### Data availability.

The genomic reads generated here were deposited in the Sequence Read Archive under project accession number PRJNA493061.
