# Supplementary figures and images for: Cryptococcus deuterogattii VGIIa Infection Associated with Travel to the Pacific Northwest Outbreak Region in an Anti-Granulocyte-Macrophage Colony-Stimulating Factor Autoantibody-Positive Patient in the United States
Source: mBio. 2019 Feb 12;10(1):e02733-18. doi: 10.1128/mBio.02733-18 (PMC6372798; doi:10.1128/mBio.02733-18)

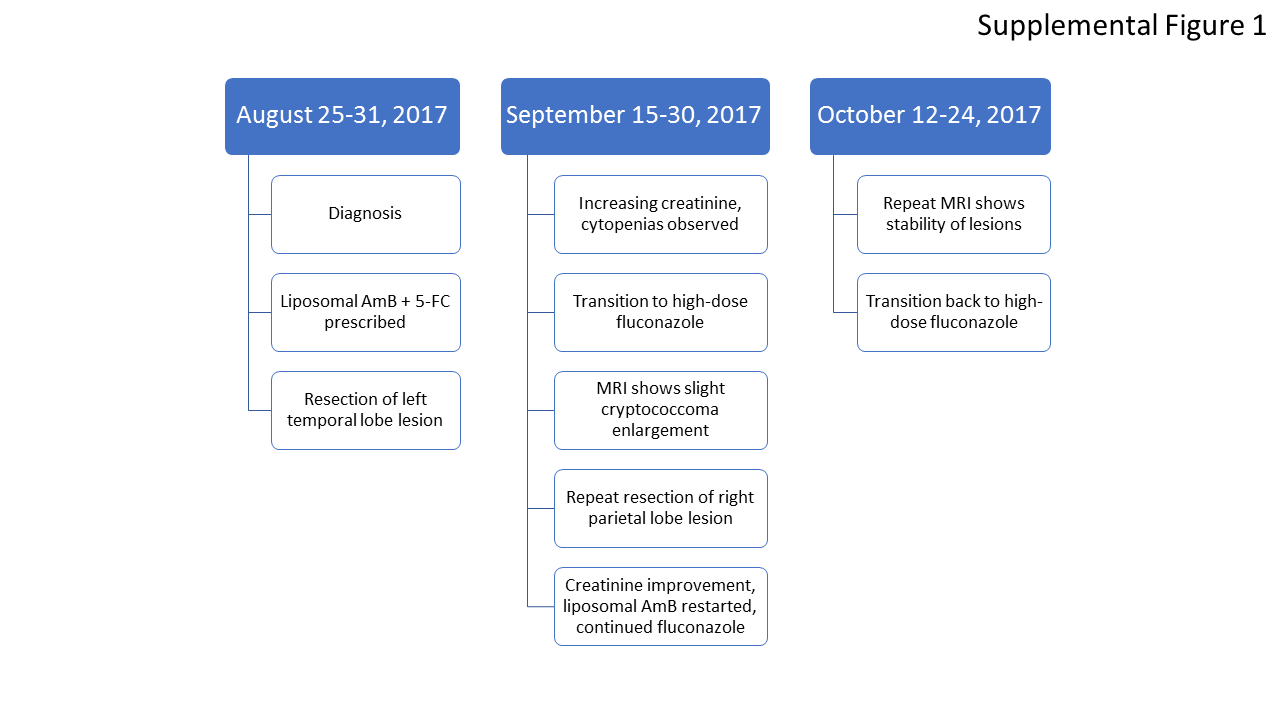

Supplement: FIG S1 [file mBio.02733-18-sf001.tif]

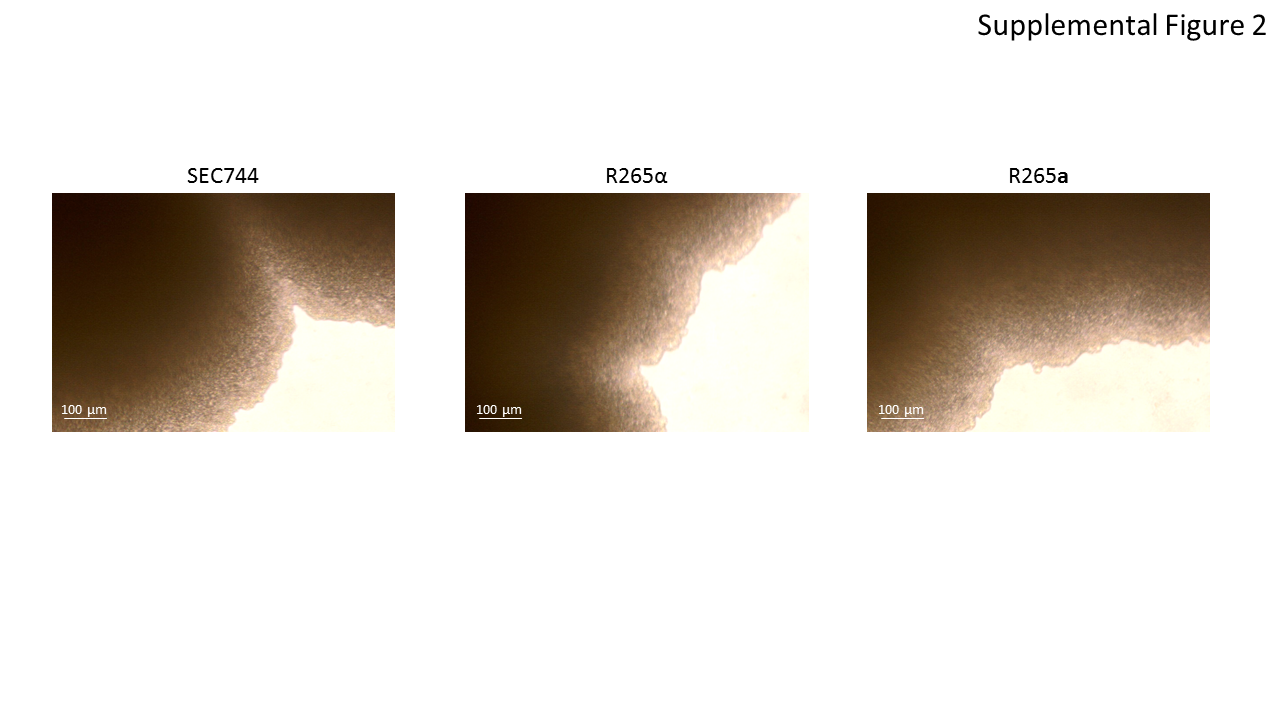

Supplement: FIG S2 [file mBio.02733-18-sf002.tif]

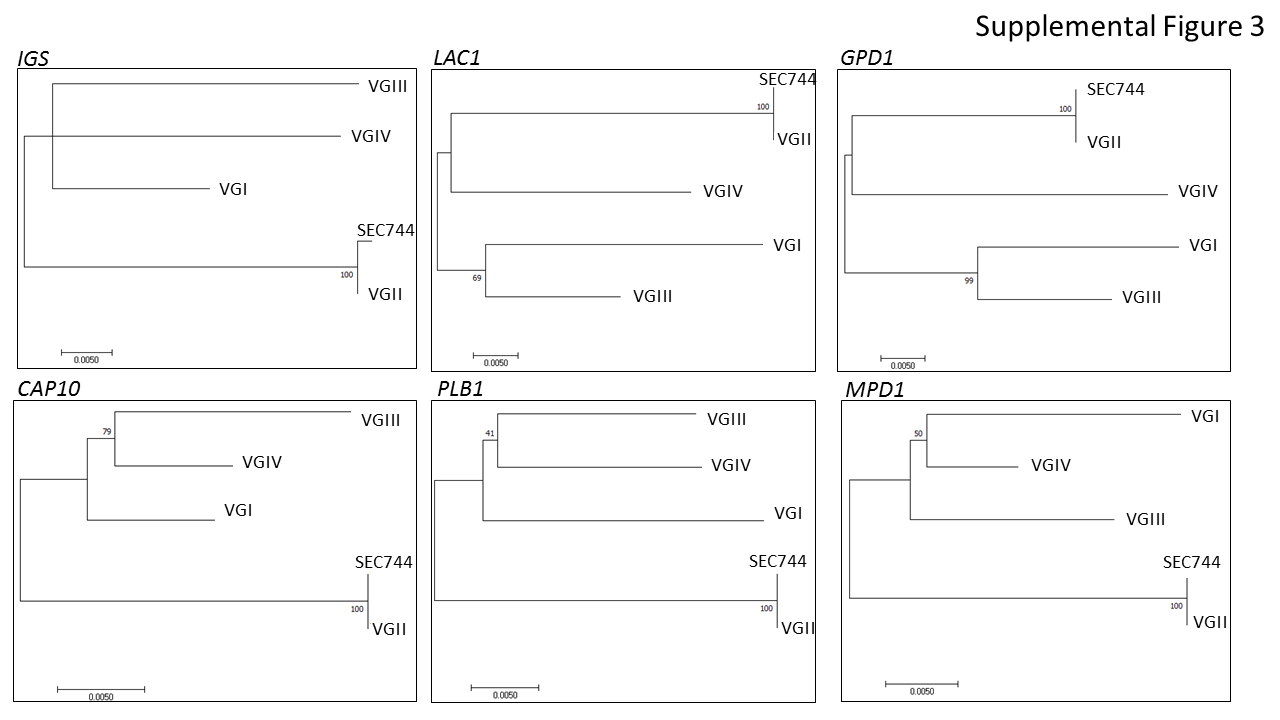

Supplement: FIG S3 [file mBio.02733-18-sf003.tif]
