# Supplementary material for: Cryptococcus deuterogattii VGIIa Infection Associated with Travel to the Pacific Northwest Outbreak Region in an Anti-Granulocyte-Macrophage Colony-Stimulating Factor Autoantibody-Positive Patient in the United States
Source: mBio. 2019 Feb 12;10(1):e02733-18. doi: 10.1128/mBio.02733-18 (PMC6372798; doi:10.1128/mBio.02733-18)
Supplement: TABLE S1 [file mBio.02733-18-st001.docx]

Supplemental Table 1. Primers used in this study

| Primer name | Sequence | Gene |
| --- | --- | --- |
| JOHE14115 | AGGGTACGTTTGAGGCCAGTT | *SXI1*α 5’ MLST primer |
| JOHE14116 | GAAAGCGTTGGCAAGGAATGA | *SXI1*α 3’ MLST primer |
| JOHE10453 | TGATCGCACGAGCCAAATCCC | *SXI2***a** 5’ MLST primer |
| JOHE10454 | GGCTTCCTGACAACACTTCTA | *SXI2***a** 3’ MLST primer |
| JOHE14408 | ATCCTTTGCAGACGACTTGA | *IGS* 5’ MLST primer |
| JOHE14409 | GTGATCAGTGCATTGCATGA | *IGS* 3’ MLST primer |
| JOHE14976 | GCACGCTCTTCTCGCCTTCAC | *TEF1* 5’ MLST primer |
| JOHE14977 | GTAGTCGGCGTAGGTCTCAAC | *TEF1* 3’ MLST primer |
| JOHE14968 | CCACCGAACCCTTCTAGGATA | *GPD1* 5’ MLST primer |
| JOHE14969 | CTTCTTGGCACCTCCCTTGAG | *GPD1* 3’ MLST primer |
| JOHE14970 | AACATGTTCCCTGGGCCTGTG | *LAC1* 5’ MLST primer |
| JOHE14971 | ATGAGAATTGAATCGCCTTGT | *LAC1* 3’ MLST primer |
| JOHE14386 | CCGGAACTGACCACTTCATC | *CAP10* 5’ MLST primer |
| JOHE14387 | GCCCACTCAAGACACAACCT | *CAP10* 3’ MLST primer |
| JOHE14974 | CTCTCATTGTTCGCCGCTACT | *PLB1* 5’ MLST primer |
| JOHE14975 | GGAAGCCGAGGTCTGATTTGG | *PLB1* 3’ MLST primer |
| JOHE14972 | TGCCCTGGATCCTAATGCTCT | *MPD1* 5’ MLST primer |
| JOHE14973 | ACCCAGACTGCCGCTGTCGTC | *MPD1* 3’ MLST primer |
